# Comparison of Echo Planar and Turbo Spin Echo Diffusion‐Weighted Imaging in Intraoperative MRI

**DOI:** 10.1002/jmri.29614

**Published:** 2024-10-10

**Authors:** James C. Thorpe, Stefanie C. Thust, Claire H. M. Gillon, Selene Rowe, Charlotte E. Swain, Donald C. MacArthur, Simon P. Howarth, Shivaram Avula, Paul S. Morgan, Rob A. Dineen

**Affiliations:** ^1^ Medical Physics and Clinical Engineering Nottingham University Hospitals Nottingham UK; ^2^ Radiological Sciences Academic Unit of Mental Health and Clinical Neuroscience, School of Medicine, University of Nottingham Nottingham UK; ^3^ NIHR Nottingham Biomedical Research Centre Nottingham UK; ^4^ Department of Brain Rehabilitation and Repair UCL Institute of Neurology, Queen Square London UK; ^5^ Sir Peter Mansfield Imaging Centre University of Nottingham Nottingham UK; ^6^ Radiology Nottingham University Hospitals Nottingham UK; ^7^ Neurosurgery Nottingham University Hospitals Nottingham UK; ^8^ Children's Brain Tumour Research Centre University of Nottingham Nottingham UK; ^9^ Radiology Alder Hey Children's Hospital NHS Foundation Trust Liverpool UK

**Keywords:** intraoperative MRI, diffusion, TSE diffusion, EPI diffusion, neurosurgery

## Abstract

**Background:**

Diffusion‐weighted imaging (DWI) is routinely used in brain tumor surgery guided by intraoperative MRI (IoMRI). However, conventional echo planar imaging DWI (EPI‐DWI) is susceptible to distortion and artifacts that affect image quality. Turbo spin echo DWI (TSE‐DWI) is an alternative technique with minimal spatial distortions that has the potential to be the radiologically preferred sequence.

**Purpose:**

To compare via single‐ and multisequence assessment EPI‐DWI and TSE‐DWI in the IoMRI setting to determine whether there is a radiological preference for either sequence.

**Study Type:**

Retrospective.

**Population:**

Thirty‐four patients (22 female) aged 2–61 years (24 under 18 years) undergoing IoMRI during surgical resection of intracranial tumors.

**Field Strength/Sequence:**

3‐T, EPI‐DWI, and TSE‐DWI.

**Assessment:**

Patients were scanned with EPI‐ and TSE‐DWI as part of the standard IoMRI scanning protocol. A single‐sequence assessment of spatial distortion and image artifact was performed by three neuroradiologists blinded to the sequence type. Images were scored regarding distortion and artifacts, around and remote to the resection cavity. A multisequence radiological assessment was performed by three neuroradiologists in full radiological context including all other IoMRI sequences from each case. The DWI images were directly compared with scorings of the radiologists on which they preferred with respect to anatomy, abnormality, artifact, and overall preference.

**Statistical Tests:**

Wilcoxon signed‐rank tests for single‐sequence assessment, weighted kappa for single and multisequence assessment. A *P*‐value <0.001 was considered statistically significant.

**Results:**

For the blinded single‐sequence assessment, the TSE‐DWI sequence was scored equal to or superior to the EPI‐DWI sequence for distortion and artifacts, around and remote to the resection cavity for every case. In the multisequence assessment, all radiologists independently expressed a preference for TSE‐DWI over EPI‐DWI sequences on viewing brain anatomy, abnormalities, and artifacts.

**Data Conclusion:**

The TSE‐DWI sequences may be favored over EPI‐DWI for IoMRI in patients with intracranial tumors.

**Level of Evidence:**

2

**Technical Efficacy:**

Stage 5

Intraoperative MRI (IoMRI) during brain tumor surgery offers an immediate assessment of the extent of resection and localization of residual tumor, provides updated anatomical information following brain shift relative to the preoperative anatomical images, and rapidly identifies potential surgical complications such as intracranial haemorrhage.^1^, ^2^ Therefore, IoMRI facilitates maximal safe tumor resection, reducing the need for early reoperation and improving overall survival.^3^, ^4^, ^5^, ^6^, ^7^

Diffusion‐weighted imaging (DWI) is a widely used MRI sequence that highlights and quantifies the level of water diffusion in a local area.^8^, ^9^ In clinical practice, DWI is widely used for the detection of acute cerebral ischemia, to which it is highly sensitive, and also has applications in identifying intracranial purulent infection.^10^, ^11^, ^12^, ^13^, ^14^ In intraoperative applications, DWI serves to identify local tissue ischemia, either in the form of mechanical retraction effects or through arterial infarction, some of which are not entirely unavoidable consequences of tumor devascularization.^15^ Beyond this, DWI can exclude distant embolic ischemia in the perioperative timeframe.^16^ In neuro‐oncology, DWI plays a supportive role in evaluating tissue cellularity and may therefore be valuable for mapping of suspected tumor residuums.^17^

Echo planar imaging (EPI) is typically used for DWI as its rapid acquisition time minimizes bulk motion artifacts.^8^ However, magnetic field inhomogeneity causes difficulties with standard EPI‐DWI, leading to susceptibility artifacts such as image distortion and signal heterogeneity (Fig. 1).^18^ These artifacts are particularly prevalent at air‐tissue boundaries where the magnetic field is distorted, which presents an increased challenge in IoMRI as air can be introduced intracranially during surgery. Pins used to hold the head during surgery also give rise to local magnetic field inhomogeneities and spatial distortions. This limits evaluation of acute tissue changes around the surgical cavity or other sites intracranially where air collects such as in the basal cisterns or ventricles. One area of particular concern is that when used intraoperatively, EPI‐DWI may give false negatives when assessing hyperacute infarction.^19^

An alternative to EPI‐DWI is turbo spin echo (TSE)‐DWI, where the refocusing radiofrequency pulses correct for macroscopic magnetic field inhomogeneities including those introduced by air‐tissue boundaries.^20^ Previously slower acquisition times (due to lower available signal requiring greater signal averaging) were an obstacle to TSE‐DWI being used clinically as physiological motion introduced unacceptable image artifacts.^8^ However, recent developments in commercially available TSE‐DWI sequences to correct for physiological motion now make this viable (Fig. 1).^21^, ^22^, ^23^ Use of TSE‐DWI has become widespread for clinical imaging of cholesteatoma because of the minimal spatial distortions induced by the air and bone of the skull base compared to EPI‐DWI.^24^

A head‐to‐head comparison in a large series has not been performed. Our clinical dataset allows a retrospective analysis to compare the image quality of EPI‐ and TSE‐DWI images acquired during IoMRI in terms of spatial distortion, detection of abnormalities, and radiologist preference.

Against this background, we aimed to compare via single‐ and multisequence assessment EPI‐DWI and TSE‐DWI in the IoMRI setting to determine whether there is a radiological preference for either sequence.

**Figure 1:**
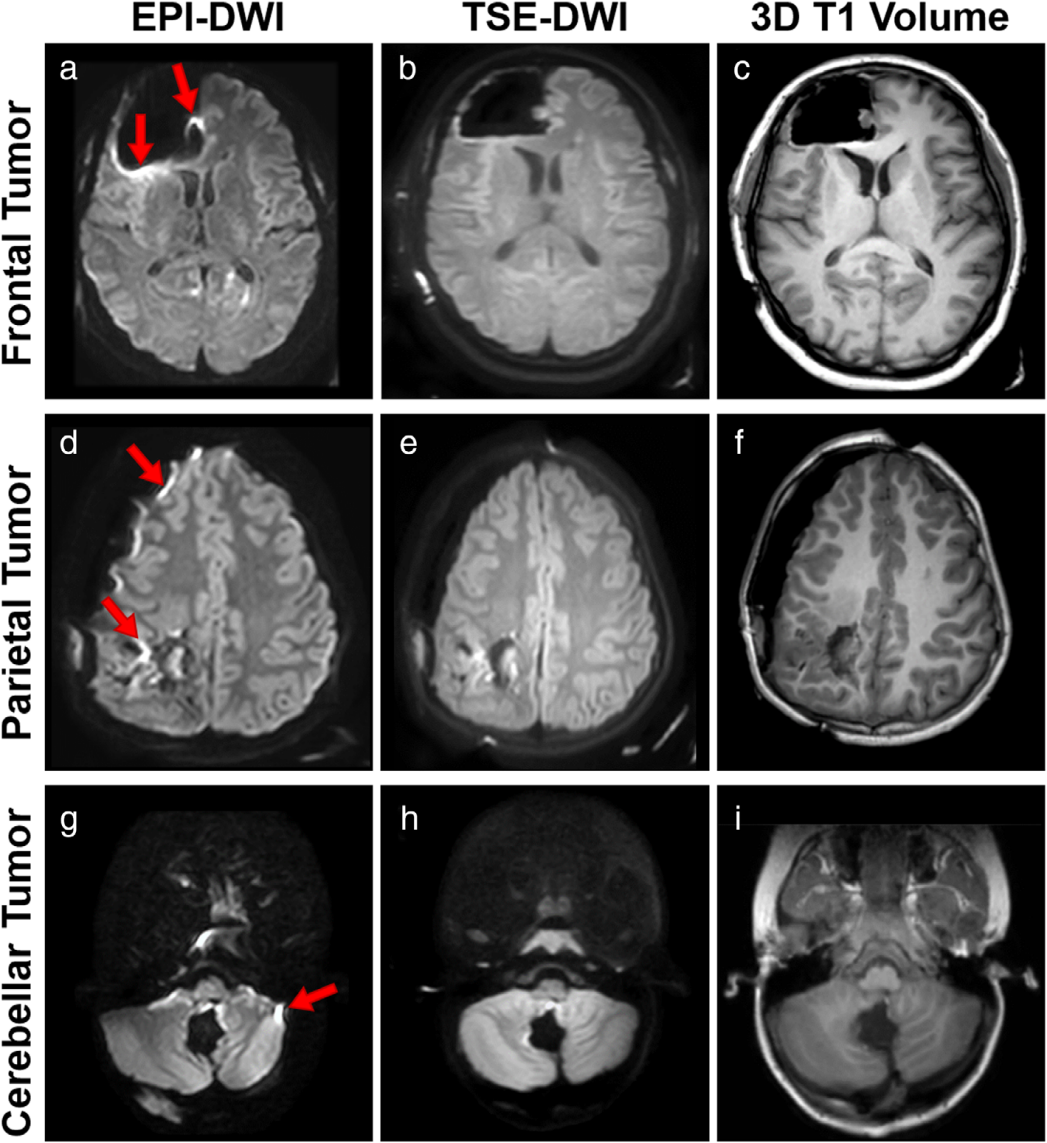
Comparison between b = 1000 EPI‐DWI (**a**, **d**, and **g**), b = 1000 TSE‐DWI (**b**, **e**, **h**), and 3D T1 volume (**c**, **f**, **i**) of IoMRI patients with frontal lobe (a–c), parietal (d, e, f) and cerebellar (g, h, i) resection with signal pile up artifact highlighted with red arrows.

## Materials and Methods

This analysis was performed as part of an ongoing service evaluation project with retrospective use of existing routine clinical data, and as such does not require Research Ethics Committee review and the requirement for consent was waived according to the UK Health Research Authority *Defining Research* table (UK Health Research Authority. Defining Research Table. https://www.hra-decisiontools.org.uk/research/docs/DefiningResearchTable_Oct2022.pdf).

### Participants

**Figure 2:**
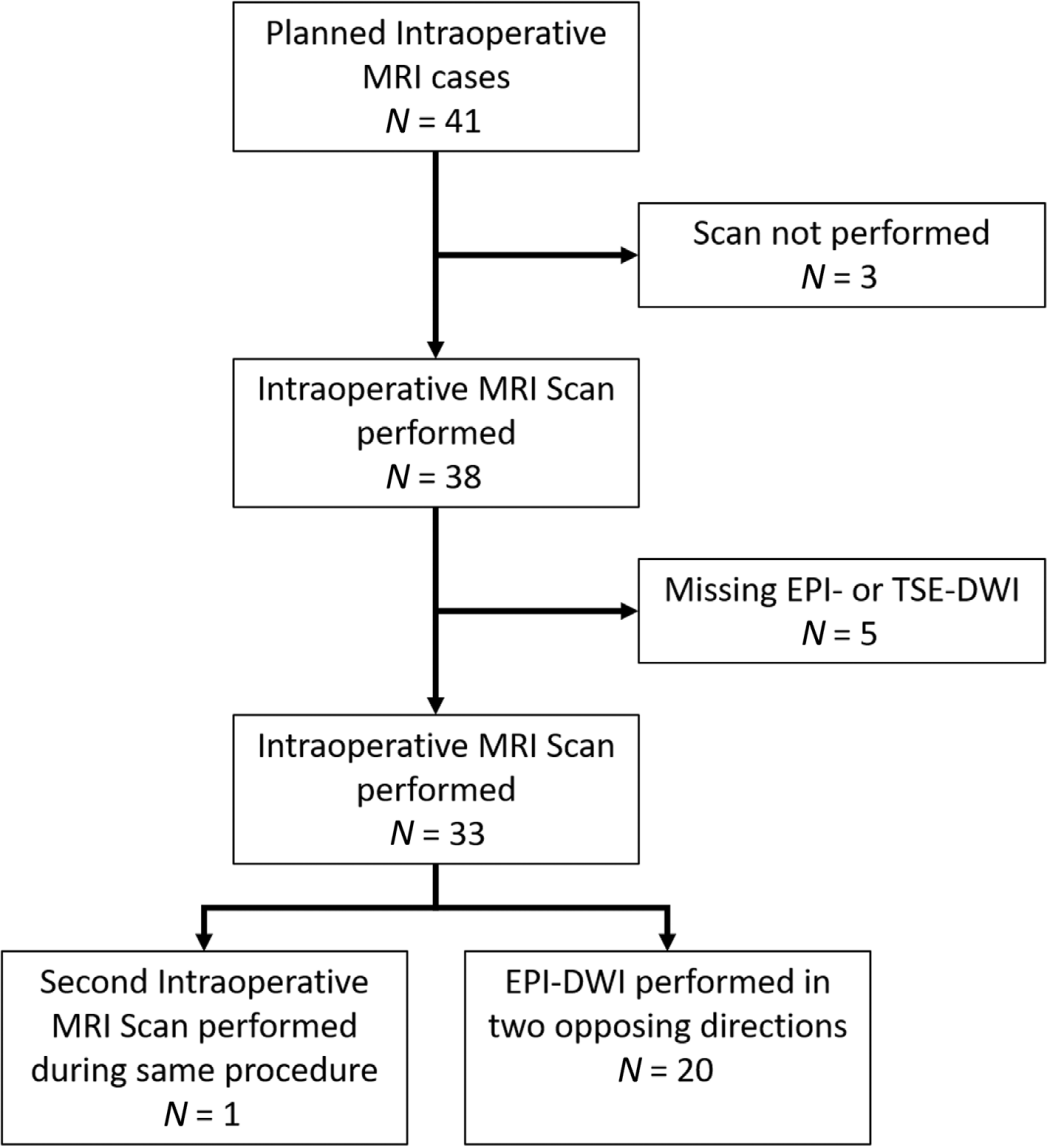
Flow chart of the patient cohort.

Between August 2021 and June 2023, 41 consecutive patients were planned for IoMRI during surgical resection of intracranial tumors. Inclusion criteria were any patient undergoing IoMRI during surgical resection of intracranial tumors scanned with both EPI‐DWI and TSE‐DWI. Exclusion criteria were any patient who did not undergo IoMRI during surgery or not scanned with both EPI‐DWI and TSE‐DWI. A total of 33 patients (11/22 male/female, 2–61 years old, median age of 8 years, 24 patients under 18 years) underwent IoMRI during surgical resection of intracranial tumors, with a clinical MRI protocol including both EPI‐DWI and TSE‐DWI. One patient had two intraoperative scans during the same tumor resection with EPI‐ and TSE‐DWI acquired for both. These two scans were treated independently resulting in a total of 34 eligible patient scans. A flow chart of the patient cohort is shown in Fig. 2. A summary of the multiple different types of tumors and locations that were included is given in Table 1.

**Table 1:** Summary of Tumor Locations and Types

Case	Tumor Location	Histological Type
1	Right frontal lobe	Oligodendroglioma
2	Posterior fossa	Pilocytic astrocytoma
3	Right temporal lobe	High grade glioma
4	Posterior fossa	Pilocytic astrocytoma
5	Posterior fossa	Pilocytic astrocytoma
6	Right temporal lobe	Ependymoma
7	Posterior fossa	Pilocytic astrocytoma
8	Right prepontine/Right suprasellar cistern	Ependymoma
9	Right frontal lobe	Oligodendroglioma
10	Posterior fossa	Ependymoma
11	Right posterior temporal lobe	Pilocytic astrocytoma
12	Right temporal lobe	Ganglioglioma
13	Left frontal lobe	Dysembryoplastic neuroepithelial tumor
14	Occipital lobe	Pilocytic astrocytoma
15	Intraventricular	Ependymoma
16	Right Parietal	Glioneuronal tumor
17	Right Parietal	Glioneuronal tumor
18	Frontal lobe	High grade glioma
19	Posterior fossa	Ependymoma
20	Posterior fossa	Ependymoma
21	Posterior fossa	Ependymoma
22	Right temporal lobe	Ganglioglioma
23	Right frontal lobe	Oligodendroglioma
24	Third ventricle	Choroid plexus papilloma
25	Posterior fossa	Pilocytic astrocytoma
26	Posterior fossa	Medulloblastoma
27	Posterior fossa	Medulloblastoma
28	Posterior fossa	Pilocytic astrocytoma
29	Temporal lobe	Dysembryoplastic neuroepithelial tumor
30	Posterior fossa	Medulloblastoma
31	Right Parietal	Glioneuronal tumor
32	Posterior fossa	Ependymoma
33	Left Parietal	Ganglioglioma
34	Posterior fossa	Medulloblastoma

### Image Acquisition

On the basis that TSE‐DWI should perform well in the IoMRI setting to provide DWI contrast with minimal spatial distortion, this sequence was included in our standard IoMRI protocol, in addition to standard EPI‐DWI, from the time that our service commenced in August 2021. All images were acquired using a Philips Ingenia Elition X 3.0 T MRI scanner (Philips Medical, Best, The Netherlands).

The EPI‐DWI images were acquired with the following scan parameters (with slight variation based on patient size): repetition time (TR) = 5727 msec, echo time (TE) = 92 msec, acquisition pixel size = 2 mm × 2 mm, interpolated pixel size = 1 mm × 1 mm, slice thickness = 3 mm, number of slices = 45, matrix size = 240, averages = 1, parallel imaging sensitivity encoding for fast MRI (SENSE) factor = 2, acquisition time = 1 hour, 9 minutes. The TSE‐DWI images were acquired with the following scan parameters (with slight variation based on size of resection cavity): TR = 3375 msec, TE = 65 msec, acquisition pixel size = 1.8 mm × 2 mm, interpolated pixel size = 1 mm × 1 mm, slice thickness = 4 mm, number of slices = 18, matrix size = 320, averages = 4, parallel imaging SENSE factor = 2, acquisition time = 4 hours, 30 minutes.

Other imaging acquired during each intraoperative case typically included T1‐weighted pre‐ and postgadolinium, T2‐weighted axial, fluid‐attenuated inversion recovery, and susceptibility‐weighted imaging.

On 20 occasions, EPI‐DWI was acquired a second time with the phase encoding direction reversed in order to allow offline quantitative evaluation of image distortion. Prior to transfer for scan it is the surgical team's usual practice to fill the operative cavity with irrigation fluid and carry out a temporary skin closure with tacking stitches. This is done to eradicate as much air introduced by surgery as possible and create a scanning environment as close to preop and postop scans as possible to minimize distortion for air‐tissue boundaries.

### Calculation of Image Distortion in EPI‐DWI

For participants with the additional reversed phase‐encoded EPI‐DWI acquisitions, image distortion was estimated by using FMRIB FSL topup version 5.0.9 (Oxford University, UK)^25^ to calculate a B_0_ field map, defining a 1 cm diameter spherical region of interest (ROI) centered on the largest field distortion in the resection cavity on the field map, and calculating the mean pixel distortion for each case where this was available. ROI placements were performed by physicist with 10 years of experience (J.C.T.). No equivalent quantitative test was performed on the TSE‐DWI as no measurable distortion was expected.

### Blinded Semi‐Quantitative Evaluation of Image Distortion

The EPI‐ and TSE‐DWI data for every case were de‐identified, randomly ordered, and rated using and ordinal scale for spatial distortion and visibility of lesions by a three neuroradiologists with 18, 12, and 4 years of experience (RAD, SCT, and CHMG, respectively) blinded to acquisition type, participant details, and additional IoMRI image sequences using the criteria given in Table 2. Review of DICOM® (Digital Imaging and Communications in Medicine) images was conducted on the b = 0 and b = 1000 images without offline distortion correction using RadiAnt DICOM viewer (Medixant, Poznan, Poland).^26^

**Table 2:** Scoring Criteria for Isolated Image Scoring by Blinded Neuroradiologist

Severity of distortions affecting the views of the resection cavity and margin	1— None	2— Minor	3— Moderate	4— Severe	5— Uninterpretable
Severity of distortions remote to the resection cavity and margin	1—None	2—Minor	3—Moderate	4—Severe	5—Uninterpretable
Severity of pileup artifacts affecting the views of the resection cavity and margin	1—None	2—Minor	3—Moderate	4—Severe	5—Uninterpretable
Severity of pileup artifacts remote to the resection cavity and margin	1—None	2—Minor	3—Moderate	4—Severe	5—Uninterpretable
Visibility of possible true diffusion abnormalities adjacent to the resection cavity	1—Very poor	2—Poor	3—Adequate	4—Clear	5—Very clear
Confidence that possible true diffusion abnormalities adjacent to the resection cavity are genuine	1 – Very unconfident	2 – Unconfident	3 – Equivocal confidence	4 – Confident	5 – Very confident

### Multiparametric Comparison of DWI Evaluation of Image Quality and Radiologist Preference

Subsequently, a visual rating was performed independently by the same three neuroradiologists with 18, 12, and 4 years of experience (R.A.D., S.C.T., and C.H.M.G., respectively) blinded to patient details and to each other's results. This rating compared EPI‐ and TSE‐DWI in context to the entirety of IoMRI sequences performed to emulate typical neuroradiologist practice at the time of intraprocedural review. The criteria used for this comparative radiological scoring was completed according to six questions. The first four questions were as follows:

1. Which DWI sequence displays overall anatomy more clearly?
2. Which DWI sequence displays abnormalities more clearly?
3. Which DWI sequence displays fewer artifacts/distortion?
4. Which DWI sequence do you prefer overall from a radiological perspective for reviewing IoMRI cases?

The four items were scored based on the radiologist's preference between the images as using a categorical scale: strong EPI preference, moderate EPI preference, weak EPI preference, no preference, weak TSE preference, moderate TSE preference, and strong TSE preference. Two further questions were as follows:

5 Does one DWI sequence display clinically important information that is not visible in the other sequence that aids your assessment?
6 Does one DWI sequence display artifacts/misleading information that is not present in the other sequence that hinders your assessment?

With the options: “Yes—EPI,” “Yes—Both,” “Yes—TSE,” and “No.” A “Free Comments” section was included to allow the radiologists to comment on anything of particular note. These comments were reviewed to identify any common comments that might allow artifacts and other image quality issues to be typified.

Finally, for each case the next follow‐up time point MRI was reviewed by three radiologists to identify evidence for any ischemic infarcts from the surgery. The findings were then compared with the initial IoMRI DWI scan findings (of both types) to determine whether any infarcts had been missed in the initial assessment.

### Statistical Analysis

Wilcoxon signed‐rank tests were used to compare the scores given for each paired case for all radiologists. A *P*‐value <0.001 was considered statistically significant difference between the two DWI sequences for a given criterion. Agreement between the three radiologists was evaluated using a quadratic weighted kappa for each pair of radiologists. The ranges of evaluation proposed by Landis and Koch^27^ were used: ≤0 = poor, 0.01–.20 = slight, 0.21–.40 = fair, 0.41–.60 = moderate, 0.61–.80 = substantial, and 0.81–1 = almost perfect. Statistical analysis was performed using SPSS 17 (SPSS Inc., Chicago, IL, USA).

## Results

### Calculation of Image Distortion in EPI‐DWI

A mean distortion around the resection cavity of 3.76 mm (*σ* = 1.71 mm) was calculated for the 20 cases in which EPI‐DWI was acquiring with two opposite phase‐encoding directions.

### Blinded Semi‐Quantitative Evaluation of Image Distortion

**Figure 3:**
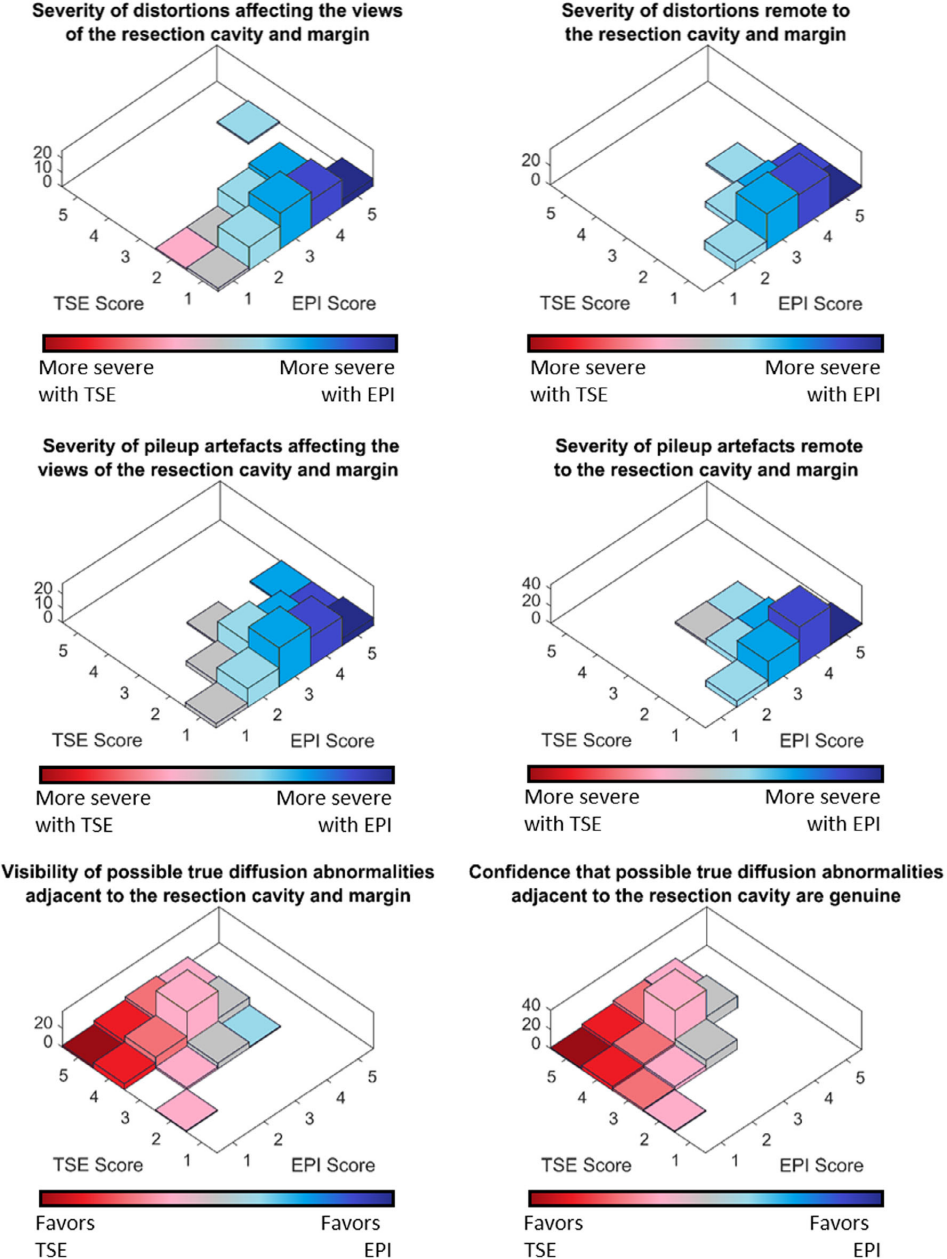
Results of blinded scoring of image quality for EPI‐DWI and TSE‐DWI.

The combined results of the blinded scoring from all three reviewers for each question are shown in Fig. 3. Each pairing of the three radiologists demonstrated substantial agreement with *κ*_W_ = 0.77, *κ*_W_ = 0.76, and *κ*_W_ = 0.80 (Rad1‐Rad2, Rad2‐Rad3, and Rad1‐Rad3, respectively). It can be seen that the severity of distortions and signal pileup artifacts both around and remote to the resection cavity and margin were scored as equal to or worse for the EPI‐DWI than the TSE‐DWI in every case for every question (the TSE‐DWI was found to be statistically significantly greater than the EPI‐DWI for all questions) with two exceptions. There were two occasions where two separate radiologists scored the TSE‐DWI slightly worse than the EPI‐DWI. In 335 out of 408 scores (across the first four questions) the EPI‐DWI sequence can be seen to have moderate or severe distortion and pileup artifacts while in 400 out of 408 scores the TSE‐DWI had no, or minor, artifact or distortion, around the resection cavity and margin. In one case moderate distortion was seen in the TSE‐DWI which can be attributed to the presence of a programmable shunt.

The visibility of possible true diffusion abnormalities and the confidence that these abnormalities are genuine was found to be at least as good, but typically greater, for the TSE‐DWI over the EPI‐DWI sequences. The scores showed that in 82 out of 102 scores, the radiologists were confident about diffusion abnormalities being genuine for TSE‐DWI sequences in isolation, but generally not confident in findings for the EPI‐DWI sequences (81 out of 102 scored equivalent confidence or below). There were insufficient cases of abnormalities remote to the resection cavity to report on the visibility and confidence of these.

### Multiparametric Comparison of DWI Sequences for Qualitative Evaluation of Image Quality and Radiologist Preference

**Figure 4:**
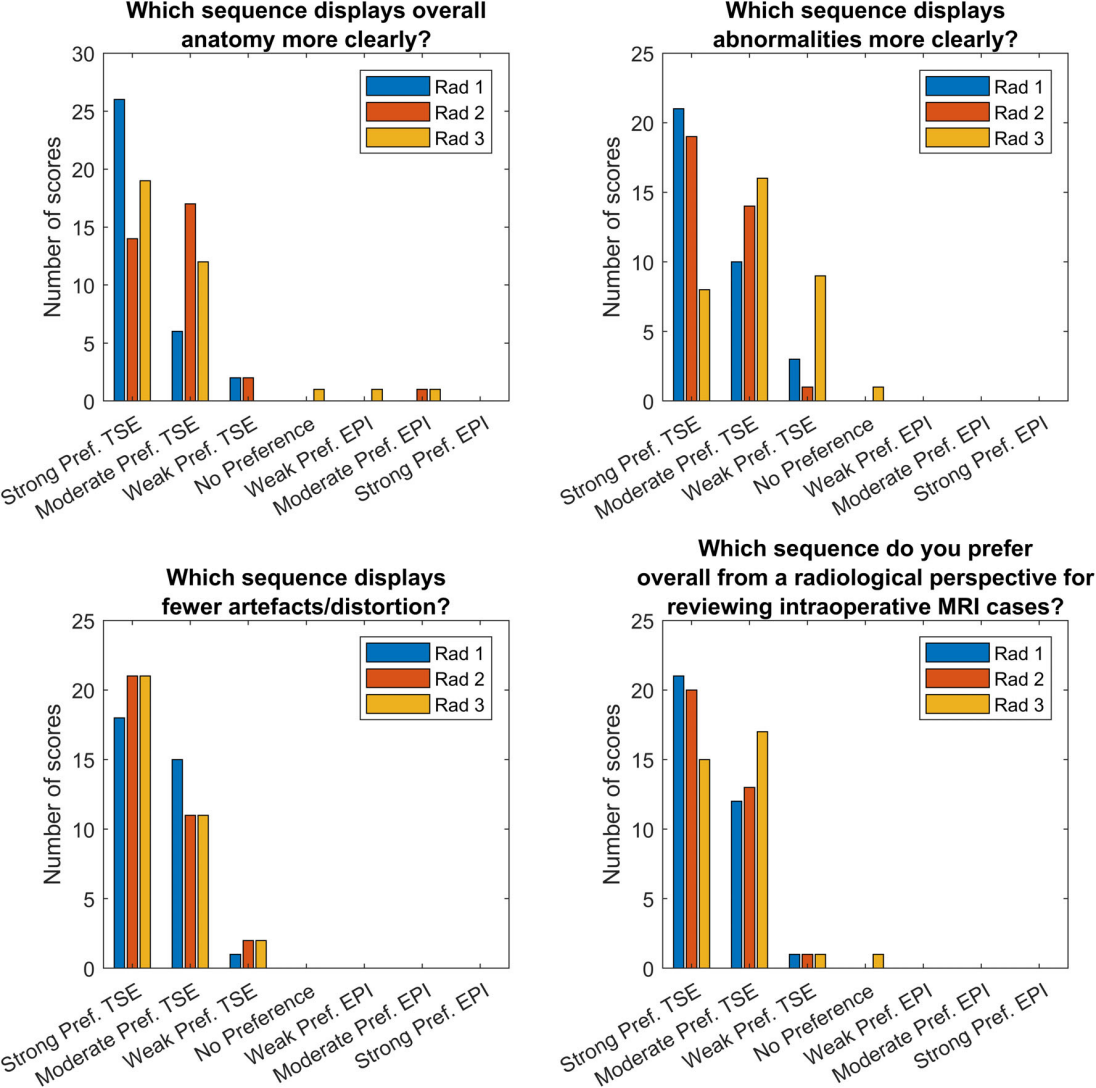
Summary of the number of scores allocated to each preference for each criterion by three radiologists.

The results of the radiological multiple sequence comparison, in which two radiologists evaluated the entire IoMRI series for each case for preference, is shown in Fig. 4. Each pairing of the three independent radiologist assessments demonstrated an almost perfect interrater agreement of *κ*_W_ = 0.94, *κ*_W_ = 0.91, and *κ*_W_ = 0.91 (Rad1‐Rad2, Rad2‐Rad3, and Rad1‐Rad3, respectively). In almost all cases both radiologists preferred the TSE‐DWI to the EPI‐DWI sequences for all four criteria, with three exceptions where a preference for EPI‐DWI was expressed, and three occasions where no preference was expressed. Notably, the most common overall answer (*N* = 21/20/15) to the question “Which sequence do you prefer overall from a radiological perspective for reviewing IoMRI cases?” was a strong preference for TSE‐DWI for all radiologists.

**Figure 5:**
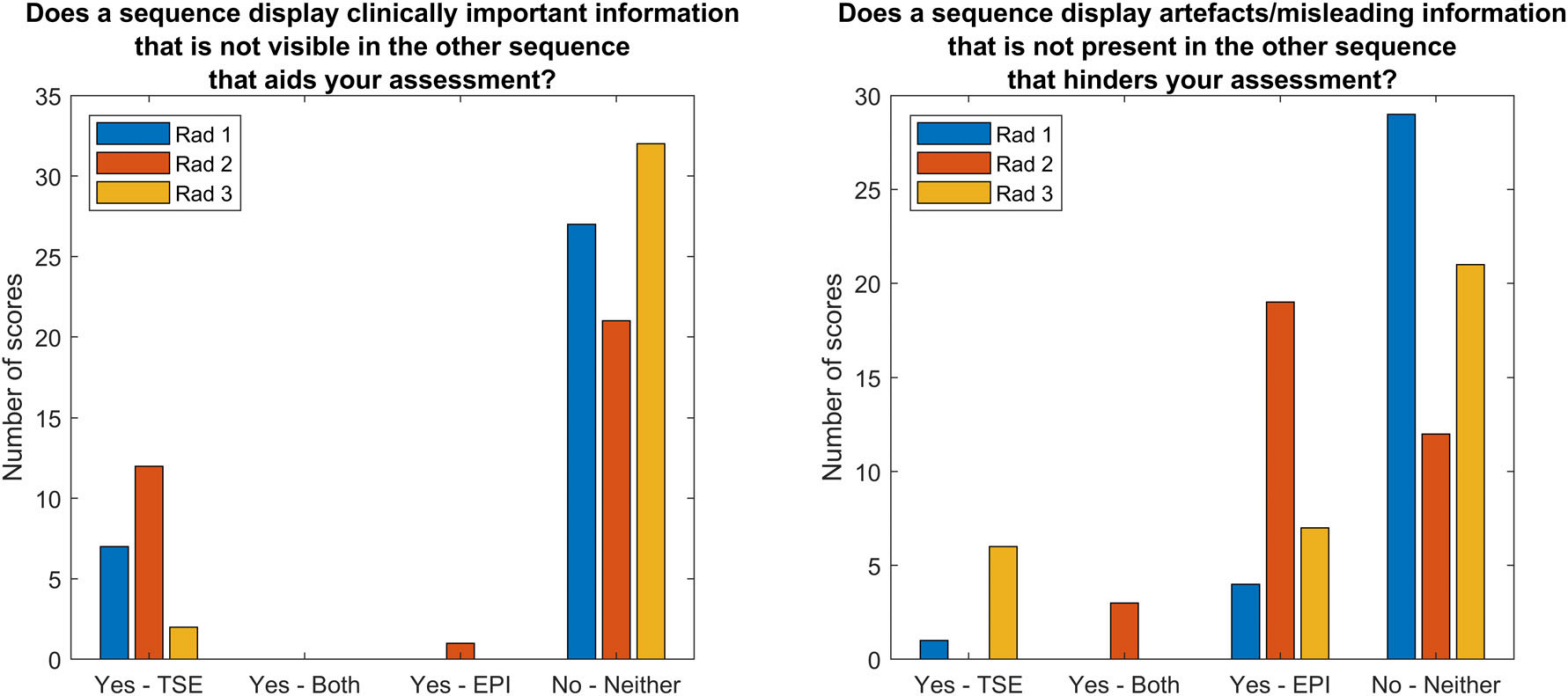
Radiologist responses to whether one sequence had clinically important/misleading information present that was not seen in the other.

**Figure 6:**
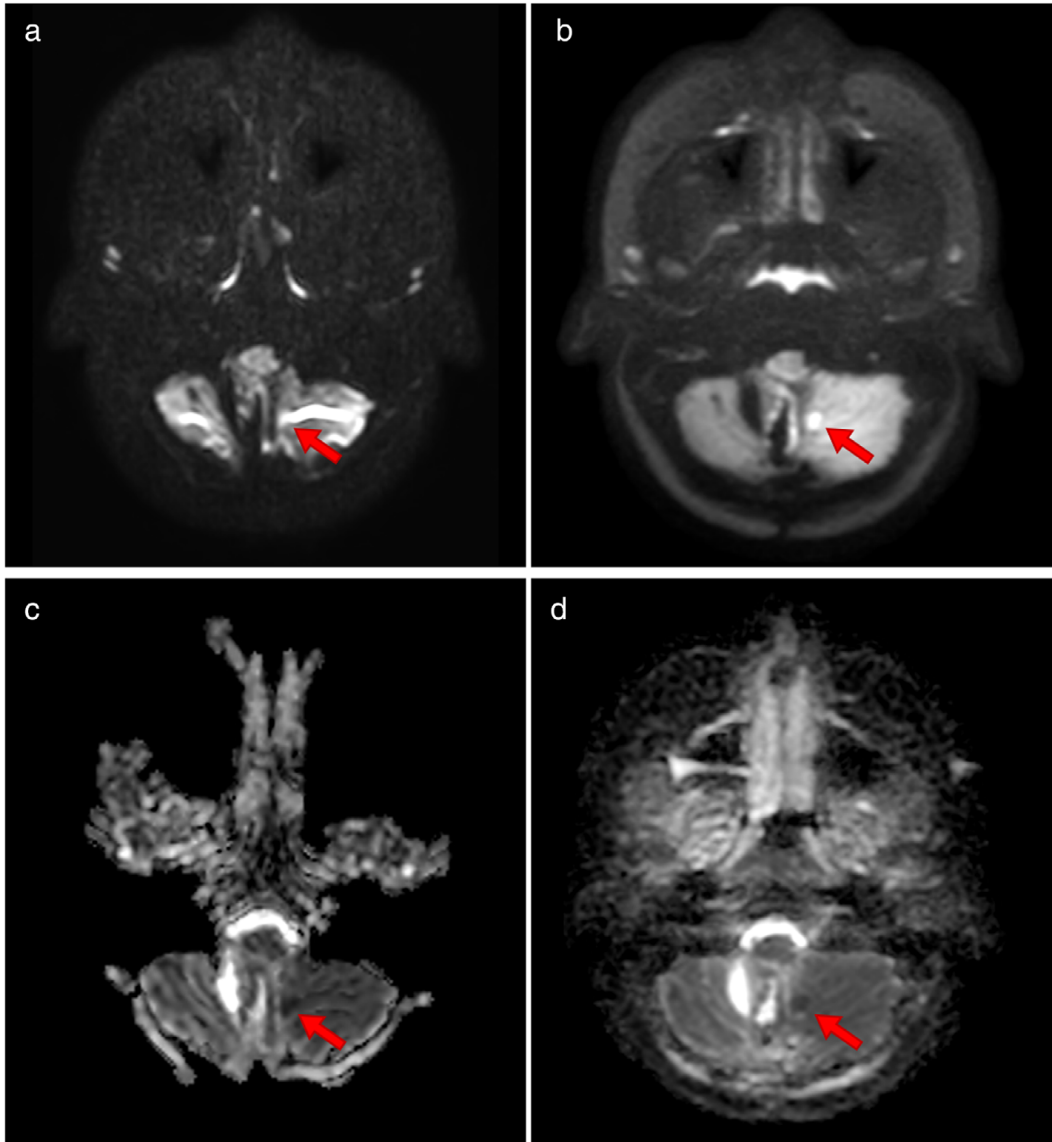
b = 1000 (**a**, **b**) and ADC (**c**, **d**) images for EPI‐DWI (a, c) and TSE‐DWI (b, d) featuring cerebellar infarct indicated by red arrow.

The radiologists were also asked whether there was any clinically relevant or misleading information present in only one of the two images. The most common reply to this answer was “No—neither” (*N* = 27/21/32 for clinically relevant, *N* = 29/12/26 for misleading) with the responses given in Fig. 5. It can be seen that all radiologists reported several occasions (*N* = 7/12/2) in which there was clinically relevant information visible in the TSE‐DWI that was not visible in the EPI‐DWI sequences, with one occasion of the EPI‐DWI containing clinically relevant information that was not present in the TSE‐DWI. Free comments on this included general comments on artifacts due to gas and blood products, and one reference to an infarct being visible in the TSE‐DWI but masked by signal pile up artifact in the EPI‐DWI. The images for this latter comment are shown in Fig. 6 which shows the b = 1000 images (Fig.1a, b) and ADC maps (Fig. 1c, d) for both EPI‐ (Fig. 1a, c) and TSE‐DWI sequences (Fig.1b, d). The large signal pileup artifact in the EPI‐DWI obscured the infarct which is clearly visible in the TSE‐DWI. With regard to artifacts and misleading information, all radiologists reported several instances (*N* = 4/19/7) of the EPI‐DWI hindering their assessment. There were also two occasions of both sequences reported as having unique misleading information, and two occasions where the TSE‐DWI had uniquely misleading information.

No ischemic infarcts were found to have been missed by either the EPI‐ or TSE‐DWI when compared to follow‐up scans performed at 1–40 days (interquartile range = 17).

## Discussion

Overall the results from the blinded scoring and direct comparisons showed that for IoMRI, TSE‐DWI sequences had consistently superior image quality compared to EPI‐DWI sequences over a broad range of visual criteria. This can be explained by the severity of magnetic susceptibility artifacts introduced to the EPI‐DWI sequence by the presence of intracranial air during the surgery, despite surgical practice of filling the cavity with irrigation fluid and carrying out a temporary skin closure. However, although the TSE‐DWI sequence was the preferred choice, there are still important considerations about its expected performance in a clinical IoMRI setting.

The lower signal and signal per unit time produced by TSE‐DWI necessitates a larger number of signal averages in order to produce diagnostic quality images.^28^ This increases the scan time for this sequence (TSE‐DWI typically 3 minutes longer for the cases used in this analysis).^29^ In the case of IoMRI there is no major concern about this increased time introducing motion artifacts as the patient will invariably be under general anesthetic with their head fastened to a frame. However, in an intraoperative setting it is desirable to acquire images as quickly as possible in order to allow the surgery to continue and reduce time under anesthetic conditions. A substantially longer DWI sequence is therefore not ideal.

A partial compromise for the scans acquired in this study was for the TSE‐DWI to have a smaller coverage and slightly increased slice thickness than the EPI‐DWI sequence, such that the TSE‐DWI sequence in this study aimed to cover the entirety of the resection cavity with some additional coverage either side, while the EPI‐DWI had typically full brain coverage. Despite this difference in coverage, the TSE‐DWI sequence was still consistently 3.5 times longer than the EPI‐DWI sequence. This difference would be further exacerbated if full brain coverage using TSE‐DWI were required.

The issue of image distortion in EPI‐DWI is well known such that there are various commercially available software packages to try to correct for this.^25^, ^30^ This includes FMRIB FSL topup^25^ as well as manufacturer‐specific inbuilt EPI distortion correction methods. Offline analysis techniques such as topup are not typically viable for IoMRI cases due to the time required to download and process the images when real‐time reporting is required to support surgical decision making.^31^ The MRI vendor distortion correction techniques were not evaluated as part of this study.

For routine clinical practice, the familiarity of radiologists with particular sequences needs to be considered. Specifically, EPI‐DWI is much more commonly used in clinical neuroradiological practice and hence radiologists may be much more familiar, comfortable, and confident reviewing EPI‐DWI sequences. We acknowledge that TSE‐DWI appears qualitatively different from EPI‐DWI, but our results indicating a preference for TSE among three clinical radiologists suggests this difference in appearance should not be a barrier to adoption of TSE‐DWI in clinical IoMRI practice. However, it may be sensible to have a period of familiarization for radiologists, with acquisition of both DWI types, at centers planning to change to TSE‐DWI for IoMRI applications. A future development of this work could be to investigate the viability of using TSE techniques for diffusion tensor imaging of the brain intraoperatively with reduced distortion.

### Limitations

The main limitation for this study is the small cohort size of *N* = 34. This is due to the slow rate of cases undergoing IoMRI guided surgery at this site. As this was a single site study performed on a single scanner from a single vendor there are further limitations on the generalizability of these findings.

## Conclusion

This study showed that when viewed in isolation, a TSE‐DWI sequence may have equal to or greater quality than an EPI‐DWI sequence in terms of distortion and signal pileup around and remote to the resection cavity, with greater visibility and confidence in reporting true diffusion abnormalities around the resection cavity for the TSE‐DWI sequence. In the multisequence comparison, a TSE‐DWI may be preferred overall for intraoperative assessments.
